# Smartphone-based self-monitoring in bipolar disorder: evaluation of usability and feasibility of two systems

**DOI:** 10.1186/s40345-018-0134-8

**Published:** 2019-01-04

**Authors:** Maria Faurholt-Jepsen, Emanuale Torri, Jesús Cobo, Daryoush Yazdanyar, Diego Palao, Narcis Cardoner, Olaf Andreatta, Oscar Mayora, Lars Vedel Kessing

**Affiliations:** 1 Copenhagen Affective Disorder Research Center (CADIC), Psychiatric Center Copenhagen, Rigshospitalet, Blegdamsvej 9, 2100 Copenhagen, Denmark; 2 Department of Health and Social Solidarity, Autonomous Province of Trento, Trento, Italy; 3 Mental Health Department, Parc Taulí, Institut d’Investigació i Innovació Sanitària Parc Taulí, I3PT, CIBERSAM, Sabadell, Spain; 4 Mental Health Department, Healthcare Trust of the Autonomous Province of Trento, Trento, Italy; 5 Fandazione Bruno Kessler Foundation, Trento, Italy

## Abstract

**Background:**

The aims of the present multicenter pilot study were to examine the feasibility and usability of two different smartphone-based monitoring systems (the Pulso system and the Trilogis-Monsenso system) from two IT companies in patients with bipolar disorder, developed and selected to be testes as a part of a European Union funded Pre-Commercial Procurement (the NYMPHA-MD project).

**Methods:**

Patients with bipolar disorder (ICD-10), > 18 years of age during a remitted, partial remitted or mild to moderate depressive state (HDRS-17 < 25) from Italy, Spain and Denmark were included. Patients were randomized 1:1 to the use of one of two smartphone-based monitoring systems. The randomization was stratified according to study location (Italy, Spain, Denmark) and all patients were followed for a 4 weeks study period. Usability and feasibility were evaluated using the Computer System Usability Questionnaire, and the Usefulness, Satisfaction, and Ease of use Questionnaire.

**Results:**

A total of 60 patients aged 18–69 years with bipolar disorder (ICD-10) recruited from Italy, Spain, Denmark were included—59 patients completed the study. In Denmark, the patients evaluated the Trilogis-Monsenso system with a statistically significant higher usability compared with the Pulso system. In Italy and Spain, the patients evaluated no statistically significant difference between the two systems in any of the categories, except for the usefulness category favoring the Trilogis-Monsenso system (z = 2.68, p < 0.01).

**Conclusions:**

Both monitoring systems showed acceptable usability and feasibility. There were differences in patient-based evaluations of the two monitoring systems related to the country of the study. Studies investigating the usability and feasibility during longer follow-up periods could perhaps reveal different findings. Future randomized controlled trials investigating the possible positive and negative effects of smartphone-based monitoring systems are needed.

## Background

Bipolar disorder has an estimated prevalence of 1–2% and accounts as one of the most important causes of disability worldwide (Pini et al. 2005). In clinical practice, there are major challenges in treating bipolar disorder (Kupfer et al. 2015). The clinical assessment of severity of depressive and manic symptoms relies on subjective information and clinical evaluations often conducted at fixed time intervals raising issues including potential patient recall bias. Despite current pharmacological and psychological treatment, patients with bipolar disorder suffer from mood symptoms half of their life time (Judd et al. 2003a), resulting in decreased quality of life, impaired cognitive functioning and psychosocial function (Strejilevich et al. 2013; O’Donnell et al. 2018; Rosa et al. 2012). These deleterious outcomes illustrate the large need for innovative new treatment strategies in bipolar disorder.

Recently, the WHO stated that: “The use of mobile and wireless technologies to support the achievement of health objectives (mHealth) has the potential to transform the face of health service delivery across the globe” (WHO 2011). MHealth interventions and monitoring systems have been developed and used within various medical conditions such as diabetes, asthma, cardiovascular disease, hypertension, chronic obstructive lung disease and headache (Hanlon et al. 2017). Within mental health, interventions and monitoring systems for depression, anxiety, substance abuse, eating disorder, schizophrenia and bipolar disorder have been developed (Riper et al. 2011; Beintner et al. 2012; Richards and Richardson 2012; Donker et al. 2013; Mayo-Wilson and Montgomery 2013; Faurholt-Jepsen et al. 2015; Berrouiguet et al. 2016; Berry et al. 2016; Depp et al. 2015; Hubley et al. 2016).

There is a high rate of smartphone ownership worldwide, and approximately 1/3 of the world’s adult population owns and uses a smartphone, and it has been estimated that by the year 2020, this proportion will increase to 80% (Chargeitspot.com 2016). Smartphones as a monitoring tool allow for ecological momentary assessments (EMA), reflecting the methods used to collect assessments of individual’s real-time states repeatedly over time and during naturalistic settings reducing the risk of recall bias (Shiffman et al. 2008; Wenze and Miller 2010; Aan het Rot et al. 2012; eMarketer 2014; Torous et al. 2017). Thus, smartphones extends the use of EMA beyond its classical use for self-reports and offer the opportunity to collect fine-grained data unobtrusively and outside the clinical settings (Ebner-Priemer and Trull 2009), and thus enable collection of data on daily subsyndromal mood fluctuations which have been associated with poor prognostic factors including impaired functioning, increased risk of hospitalization, high risk of relapse, and comorbid substance use disorder and personality disorders (Strejilevich et al. 2013; O’Donnell et al. 2018; Judd et al. 2003b; Bopp et al. 2010; Joffe et al. 2004; Kupka et al. 2007; Patel et al. 2015; Gershon and Eidelman 2015).

The Next Generation Mobile Platforms for Health, in Mental Disorders (NYMPHA-MD) project was a EU funded international project conducted in Denmark, Spain and Italy during the years 2015–2018. The project was funded as a project focused on the implementation of a Pre-Commercial Procurement (PCP) of mobile e-health services for supporting physicians and patients in the treatment of bipolar disorder through continuous patients monitoring to dynamically support illness management and potentially identify early deteriorations in mood and other symptoms suggesting the onset of depression or (hypo)mania. PCP as a concept provided by the EU challenges industry from the demand side to develop innovative solutions for public sector needs and it provides a first customer reference that enables companies to create competitive advantages on the European market. PCP enables public procurers to compare alternative potential solutions approaches and filter out the best possible solutions that the market can deliver to address the public need. It is an important tool to stimulate innovation as it enables the public sector to steer the development of new solutions directly towards its needs. In PCP, public procurers buy research and development services from several competing suppliers in parallel to compare alternative solution approaches and identify the best value for money solutions that the market can deliver to address their needs. PCP is split into phases (solution design, prototyping, original development and testing of a limited set of first products) with the number of competing providers being reduced after each phase. PCP can go up to the development but does not cover large scale commercialization. The EU supports PCP because it facilitates development of innovative solutions, facilitate the access the new innovative players (e.g. startups), reduce costs for procurers and create wider markets for companies and share the risks and benefits of designing, prototyping, and testing new products and services between procurers and suppliers (The European Union 2018).

Within bipolar disorder an innovative mobile e-health service approach in treatment could allow for an early intervention of mental health care professionals and a personalized and continuous feed-back to patients about warning signs and indications for referral to mental health care professionals or self-management. From this point of view, access to interactive tools able to deliver psycho-education and self-help sessions according to standardized and effective models was developed by two IT companies, where their solutions were selected among other companies’ innovative solutions during the PCP to be tested. Thus, the usability of two IT companies solutions were tested in the present multicenter pilot study. To reflect the real-life usability and feasibility of the solutions provided by two different IT companies, patients with bipolar disorder and clinicians across three different European countries evaluated these solutions. The solutions were chosen to be tested in each of the counties that the project group represented.

The monitoring model of such type of approach was based on a portable data acquisition system able to obtain continuous measurements related to their affective state, also giving feed-backs and visualizing data to patients, thus enhancing patients’ awareness and empowering attitude and supporting their self-management, with the support of mental health care professionals. The NYMPHA-MD project defined the framework of a PCP for the provisioning of next generation services advocated for mental health treatment with a specific focus on bipolar disorder based on the use of new technologies. In particular, the NYMPHA-MD project focused on a first instance in identifying the different requirements involved in the structuring of mental health services with a focus on bipolar disorder treatment in order to construct a reference model of service provisioning useful in different European contexts. This model was utilized to produce a Call for Tender for the PCP aiming to provide a set of pilot experimentations implementing mobile e-health services for bipolar disorder treatment in a real-world context and to be tested in three European countries.

The aims of the present multicenter pilot study were to examine the feasibility and usability of two different smartphone-based monitoring systems (the Pulso system and the Trilogis-Monsenso system) from IT companies selected as a part of the NYMPHA-MD project in patients with bipolar disorder in three European countries.

## Methods

During the process of the NYMPHA-MD project, two smartphone-based monitoring systems were developed by two IT companies, and their solutions were selected among other companies’ innovative solutions during the PCP to be tested. Thus, the usability of two IT companies solutions were tested in the present multicenter pilot study.

To reflect the usability and feasibility in all the three European countries involved in the NYMPHA-MD study, the NYMPHA-MD feasibility studies were conducted at all three NYMPHA partners’ location (Italy, Spain, Denmark) by local doctors, clinicians and researchers during the period from January 2018 to March 2018.

### Study participants and settings

Inclusion criteria: Patients over the age of 18 years with a bipolar disorder diagnosis according to ICD-10 using Schedules for Clinical assessments in Neuropsychiatry (SCAN) (Wing et al. 1990) were invited to participate in the NYMPHA-MD feasibility study on one of the three study locations.

Exclusion criteria: Patients who were pregnant and with a lack of local language skills (Italian, Spanish, Danish).

The feasibility studies were conducted by each NYMPHA-MD project partner at their local hospitals in Italy (Mental Health Department, Healthcare Trust of the Autonomous Province of Trento), Spain (Corporació Sanitária Parc Taulí, Mental Health Department, Sabadell, Barcelona), and Denmark (Psychiatric Center Copenhagen, Department O, Copenhagen). In Denmark patients were recruited from The Copenhagen Affective Disorder Research Center at the Psychiatric Centre Copenhagen, Rigshospitalet, Copenhagen University Hospital, which is a specialized outpatient clinic. In Spain, patients were recruited from the Affective Disorder program—a facility specialized in the treatment of inpatients and outpatients, located in the Parc Tauli University Hospital. In Italy, patients were recruited from the Community Mental Health Centre of the Mental Health Service of Trento, which is a part of the network of psychiatric services delivered by Healthcare Trust of the Autonomous Province of Trento.

### Study design

The study aimed to include 20 patients with bipolar disorder at each of the three study locations with a follow-up period of 4 weeks. Potential participants were invited to participate in the NYMPHA-MD study by contact from the NYMPHA-MD staff. All potential participants who accepted to meet with the NYMPHA-MD staff for further study information were screened by trained researchers to make sure they fulfil the criteria for inclusion and were then included in the NYMPHA-MD study. Following inclusion, baseline assessments were conducted on all patients, and after these assessments the patients were randomized 1:1 to the use of one of the two smartphone-based monitoring systems provided by the two different IT companies. The randomization was stratified according to study location (Italy, Spain, Denmark) and all patients were followed for a 4-week study period with assessment at baseline and at the end of study. Randomization was done using http://www.randomization.com.

Since the study was not a randomized controlled trial aiming to investigate the effect of a smartphone-based intervention, but the feasibility and usability of two different systems, the study was designed and powered to be able to investigate differences in symptoms and functioning due to using a smartphone-based monitoring system. The study aimed to examine and record the feasibility, and usability of two different smartphone-based monitoring systems including commercial wrist worn accelerometers monitoring the level of physical activity.

Patients included in the NYMPHA-MD study were instructed to use one of two smartphone-based monitoring systems including a wrist worn accelerometer, which were randomly assigned to daily use during a 4-week study period. The monitoring systems, smartphones and accelerometers were provided by the two IT companies selected to participate as part of the ongoing PCP NYMPHA-MD project. At all three locations, the patients could use their own smartphone during the study.

### Outcome assessments

Researchers who were blinded to the patients’ smartphone data and who were not involved in the treatment of the patients carried out all outcome assessments. The bipolar disorder diagnosis according to ICD-10 was confirmed by a SCAN interview before inclusion of the patients. The patients were, regardless of randomization group, invited for outcome assessments by researchers blinded to smartphone data. At each visit, the assessments included a clinical rating of the severity of depressive and manic symptoms using the Hamilton Depression Rating Scale (HDRS-17) (Hamilton 1967) and the Young Mania Rating Scale (YMRS) (Young et al. 1978), respectively.

All participants filled out the feasibility and usability evaluation questionnaires the Computer System Usability Questionnaire (CSUQ), and the Usefulness, Satisfaction, and Ease of use Questionnaire (USE) (Lewis 1995; Lund 2001). The CSUQ is a 19-item questionnaire concerning global overall system level usability and usability at a more detailed scenario level than the USE with scoring levels between 19 and 133. Higher scores indicate higher usability. The USE is a 30-item questionnaire concerning usefulness, ease of use, ease of learning and satisfaction with scoring levels between 30 and 210. Higher scores indicate higher ease of use.

The patient-based evaluation of the two smartphone-based systems (smartphone-based self-monitoring and wrist worn accelerometers) was conducted separately for each of the three countries (Denmark, Italy and Spain). In Denmark patients evaluated the smartphone app and the wristband with both questionnaires separately. The two systems were evaluated as a combined system (smartphone app and wristband as a app + wristband system) in Italy and Spain.

Clinicians and/or researchers also evaluated the monitoring systems. In addition, benefits, disadvantages and suggestions for improvements of the two monitoring systems from the patients were noted.

### The monitoring systems provided by two different IT companies

#### The smartphone-based monitoring systems

**Fig. 1:**
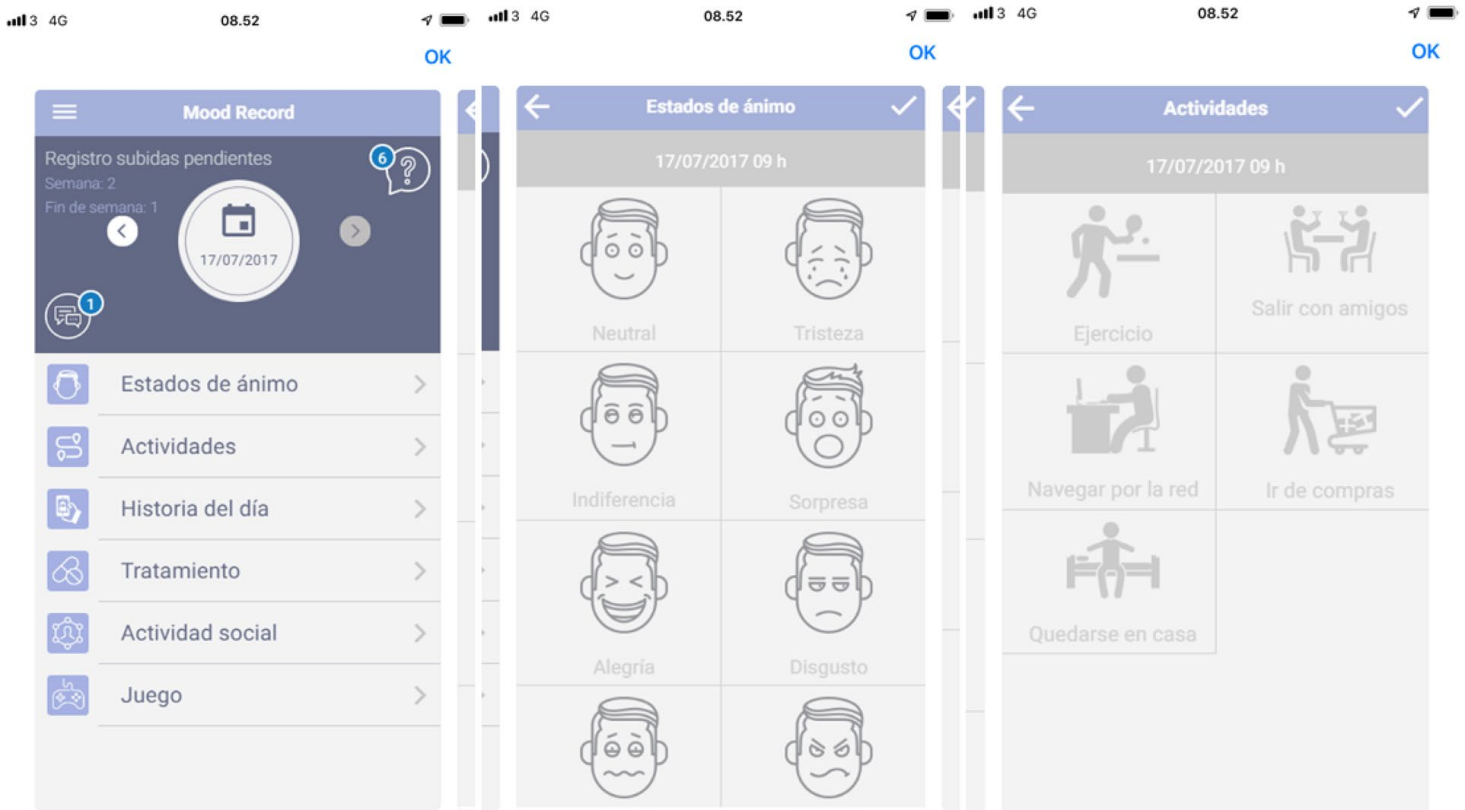
Screenshots from the Pulso monitoring system

The Pulso system: Once a day the patients should enter and evaluate mood, activity level, enter a story of the day, whether medicine was intake and activity level on social media. The mood was evaluated by choosing between eight different face icons (neutral, sadness, contempt, surprise, disgust, fear, joy and anger). The activity level was evaluated by choosing between five different activities that the patients might have been doing during the day (work out, go out with friends, surf the internet, go shopping and stay at home). A story of the day was registered by recording an audio/video file about the day with voice emotion recognition and facial recognition of emotions (acting as a diary). The registration of medicine intake was done by typing yes or no whether the medicine was taken or not. The activity on social media was registered by entering the number of likes, new friends, pictures and comments given to the various social media. In addition, the app included various psychological tests (Bus Durkee Hostility, Inventory, Beck Hopelessness Scale, Patient Health Questionnaire-15, Barrat’s Impulsiveness Scale, Percieved Stress Scale and EQ-5D-3L) that the patient could complete. Screenshots from the Pulso system are provided in Fig. 1.

**Fig. 2:**
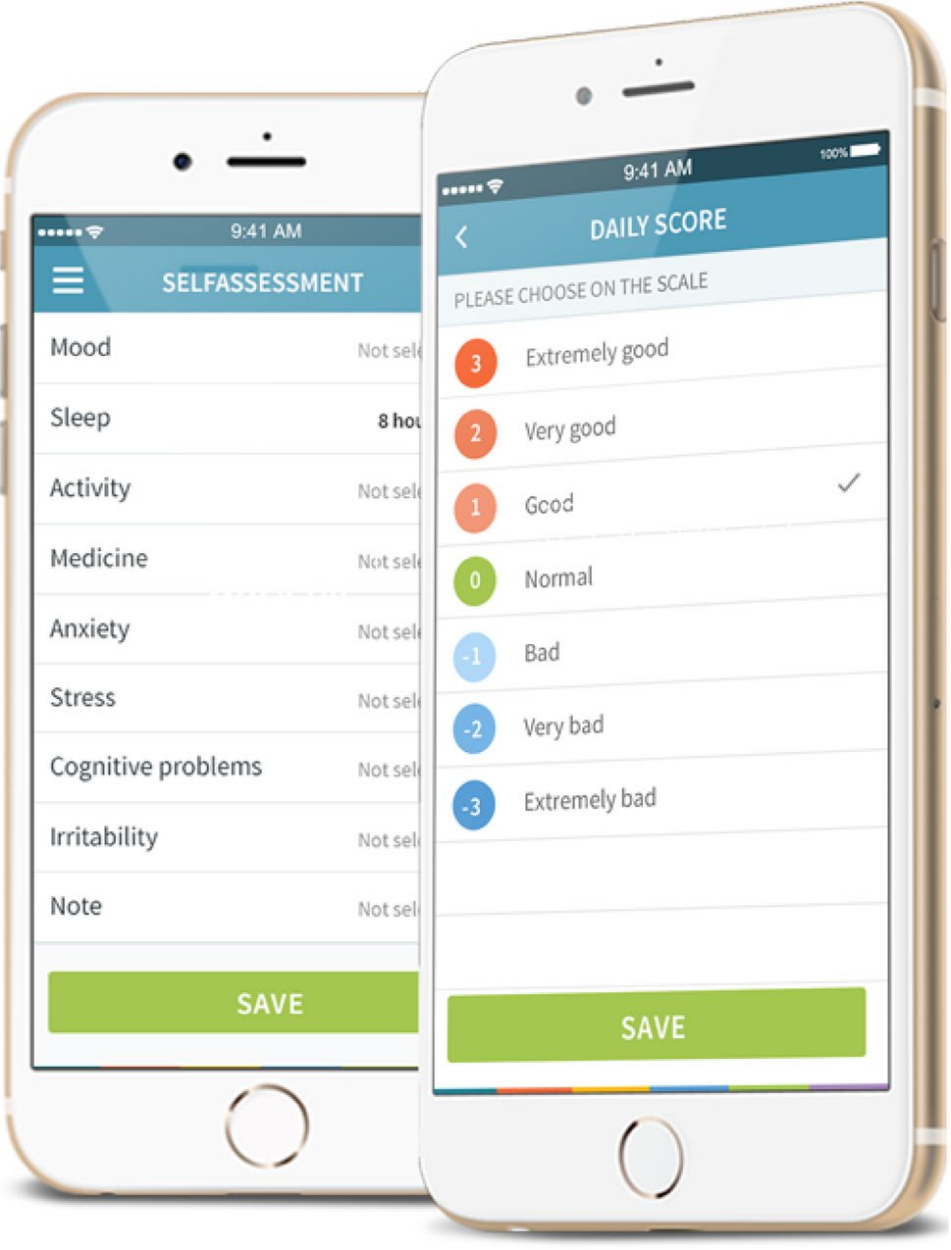
Screenshots from the Trilogis-Monsenso monitoring system

The Trilogis-Monsenso system: Once a day the patients were prompted at a self-chosen time to evaluate an overall daily score, mood, medicine intake, activity level and amount of sleep. The overall daily score was registered by choosing between seven different scores ranging from − 3 to 3 − 3 equals very bad, 0 equals neutral and 3 equals very good). The mood was evaluated by choosing between seven different scores ranging from − 3 to 3 (− 3: severe depression, 0: neutral and 3: severe mania). The registration of medicine intake was done by typing yes or no whether the medicine was taken or not. The activity level was evaluated by choosing between seven different scores ranging from − 3 to 3 (− 3: very low, 0: normal and 3: very high). The registration of amount of sleep was done by entering time of start and end of sleep from the day before. In addition, the app had the possibility to enter secondary parameters such as level of cognitive function, amount of alcohol intake, anxiety level, feelings, self-harm and drugs. It is also possible to create personalized parameters that could be important for the patients to evaluate (early warning signs and triggers). Screenshots from the Trilogis-Monsenso system are provided in Fig. 2.

#### The wrist worn accelerometers

Both IT companies provided wristworn accelerometers in addition to the smartphone-based monitoring systems. The Trilogis-Monsenso and the Pulso wristband were associated to different wristband apps and devices. The Pulso company chose to use the FitBit accelerometer. The Trilogis-Monsenso company chose to use the Nokia Health accelerometer. The wristband app was intended to measure sleep and activity (step counts). The patient got the app installed and were guided on how to use the app. Since the Trilogis-Monsenso wristband was affiliated to the Nokia Health app and the Pulso wristband was affiliated to the FitBit app, the technical specifications of the two wristbands were defined and developed by these two companies.

### Ethics

The feasibility studies applied with local ethical requirements depending on local regulations.

### Statistical analyses

Across both questionnaires, the individual items in the CSUQ and the USE questionnaires were answered with a number ranging from one to seven, where 7 indicated the highest response (‘strongly disagree’) and 1 the lowest response (‘strongly agree’). Across both questionnaires higher scores indicated higher feasibility and usability. There was also an option “NA” (“Not Able to answer”) if the patient was unable to answer the question. Answers in both questionnaire were categorized into four categories in each questionnaire. The answers from the CSUQ were categorized into the following categories: “Overall”, “System usability”, “Info quality” and “Interface quality”. The answers from the USE questionnaire were categorized into the following categories: “Usefulness”, “Ease of Use”, “Ease of learning” and “Satisfaction”. The average score of all answers from a category was found for each system: the Pulso system and the Trilogis-Monsenso system. As part of the pilot studies in Denmark, the wristband app and smartphone-based apps were evaluated separately. The average of the answers from the 3 categories added together was called “Overall”. A Mann–Whitney U-test was used to test for statistical significant differences in usability, acceptability etc. between two different apps. p-values ≤ 0.05 were considered statistically significant.

## Results

The NYMPHA-MD feasibility and usability pilot study was conducted simultaneously in Denmark, Spain and Italy from January-March 2018.

### Participants

#### Denmark

A total of 58 patients with bipolar disorder (app. 70% bipolar disorder type I and 30% bipolar disorder type II) were contacted either by SMS or by telephone regarding participation in the pilot study. Of the 58 patients with bipolar disorder, 20 patients (6 males and 14 females) were included in the NYMPHA-MD pilot study. Major reasons for declining to participate were; the patients could not see the purpose of the study, the patients didn’t want to have new apps installed on their smartphone or they did not have the time to participate. All patients were in a remitted, partial remitted or mild to moderate depressive state (HDRS-17 < 25) at inclusion and aged 21–54 years. None of the patients were hospitalized or psychotic during the study.

#### Italy

A total of 36 patients with bipolar disorder (app. 70% bipolar disorder type I and 30% bipolar disorder type II) were contacted regarding participation in the pilot study. Of the 36 patients with bipolar disorder, 20 patients (7 males and 13 females) were included in the NYMPHA-MD pilot study. Major reasons for declining to participate were; the patients only wanted to use a regular mobile phone (not smartphone), the patients did not use a mobile phone, and that they did not have the time to participate or did not feel like participating. All patients were in a remitted, partial remitted or mild to moderate depressive state (HDRS-17 < 25) and aged 18–69 years. None of the patients were hospitalized or psychotic during the study.

#### Spain

A total of 26 outpatients with bipolar disorder (app. 70% bipolar disorder type I and 30% bipolar disorder type II) were contacted regarding participation in the pilot study. Of the 26 patients, a total of 20 patients (6 males and 14 females) were included in the NYMPHA-MD pilot study. Major reasons for declining to participate were; a change in the diagnosis from the initial assessment, it was not possible to get in contact with the patients, and the patients did not have the time to participate or did not feel like participating or used an old cell phones and did not want to change phone. All patients were in a remitted, partial remitted or mild to moderate depressive state. One of the patients included did not enter self-monitoring parameters into the app at any timepoint and did not use the wristband, and finally therefore could not evaluate both. All patients were in a remitted, partial remitted or mild to moderate depressive state (HDRS-17 < 25) and aged 32–67 years. None of the patients were hospitalized or psychotic during the study.

None of the patients included were loaned a smartphone during the study.

#### Patient-based evaluation of monitoring systems

The patient-based evaluation of the two monitoring systems was conducted separately for each country (Denmark, Italy and Spain). Data from Italy and Spain could not be combined with data from Denmark, since the evaluation was conducted differently at these sites. While in Denmark patients evaluated the smartphone-based systems and the wristband system (app and device) separately in relation to each of the two questionnaires (CSUQ and USE), the two systems were evaluated as a combined system in Italy and Spain (the smartphone-based system and the wristband system). Table 1 presents summary of mean values of scores in each of the categories in each of the two questionnaires in each of the three countries.

**Table 1:** Patient-based evaluation of the two NYMPHA-MD selected monitoring systems, N = 60

	Pulso	Trilogis-Monsenso
*Denmark, N = 20*		
CSUQ overall	2.9	5.2
CSUQ system usability	3.0	5.7
CSUQ info quality	2.9	5.2
CSUQ interface quality	2.5	4.0
USE usefulness	1.9	3.8
USE ease of use	3.5	4.9
USE ease of learning	4.4	6.0
USE satisfaction	1.8	4.0
*Italy, N = 20*		
CSUQ overall	5.1	5.2
CSUQ system usability	5.5	5.7
CSUQ info quality	4.8	5.2
CSUQ interface quality	4.8	4.0
USE usefulness	3.3	3.8
USE ease of use	4.9	4.9
USE ease of learning	5.5	6.0
USE satisfaction	4.1	4.0
*Spain, N = 19*		
CSUQ overall	5.9	6.3
CSUQ system usability	5.9	6.6
CSUQ info quality	5.9	6.0
CSUQ interface quality	5.7	6.1
USE usefulness	4.5	4.9
USE ease of use	5.8	6.4
USE ease of learning	6.2	6.5
USE satisfaction	5.2	5.5

Data are mean

**Fig. 3:**
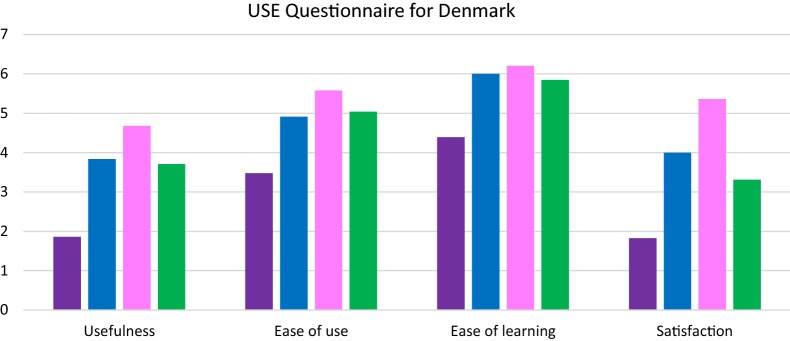
The Pulso system. The Trilogis-Monsenso. The Pulso wristband system. The Trilogis-Monsenso wristband system. Patient-based evaluation measured using the CSUQ questionnaire of the smartphone-based self-monitoring systems the Trilogis-Monsenso system and the PULSO system and the two different wristband systems

**Fig. 4:**
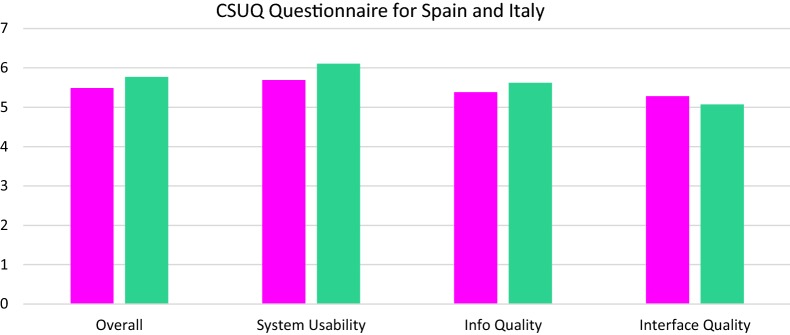
The Pulso system. The Trilogis-Monsenso. The Pulso wristband system. The Trilogis-Monsenso wristband system. Patient-based evaluation measured using the USE questionnaire of the smartphone-based systems the Trilogis-Monsenso system and the PULSO system and the two different wristband systems

Patient-based evaluation in Denmark: Regarding the smartphone-based apps, overall when compared with the Pulso system, the Trilogis-Monsenso system generally scored higher in evaluation categories using the CSUQ and USE questionnaires (Figs. 3, 4). There was a statistically significant difference in usability measures evaluated using both the CSUQ (5.66 vs. 3.04) and the USE questionnaire (3.84 vs. 1.84). In all evaluation categories measured using the CSUQ questionnaires the Trilogis-Monsenso system had statistically significant higher scores compared with the Pulso system (z > 2.58, p < 0.001).

Concerning the wristbands, the wristband system (FitBit app and device) used by the Pulso system had the highest score in overall and interface quality measured by the CSUQ questionnaire, and also scored higher in all categories in the USE questionnaire compared with the wristband system (Nokia Health app and device) used by the Trilogis-Monsenso system. The largest difference in the CSUQ questionnaire on average values between the wristband chosen by the Pulso system (FitBit) and the wristband chosen by the Trilogis-Monsenso system (Nokia Health) was in interface quality (5.83 vs. 4.72), and in the USE questionnaire in satisfaction (5.36 vs. 3.31). In the CSUQ questionnaire, there was a statistically significant difference between the wristband chosen by the Pulso system (FitBit) and the wristband chosen by the Trilogis-Monsenso system (Nokia Health) in overall, system usability and interface quality (z = 3.26, p < 0.01; z = 2.77, p < 0.01; z = 2.15, 0.01 < p < 0.05), but there was no statistically significant difference in information quality (z = 0.42, p = 0.68). In the USE questionnaire, there was a statistically significant difference between Fitbit and Nokia Health in usefulness and satisfaction (z = 2.90, p < 0.01; z = 5.50, p < 0.01) but not in ease of use and ease of learning (z = 1.88, p = 0.06; z = 0.43, p = 0.67) (Figs. 3, 4).

**Fig. 5:**
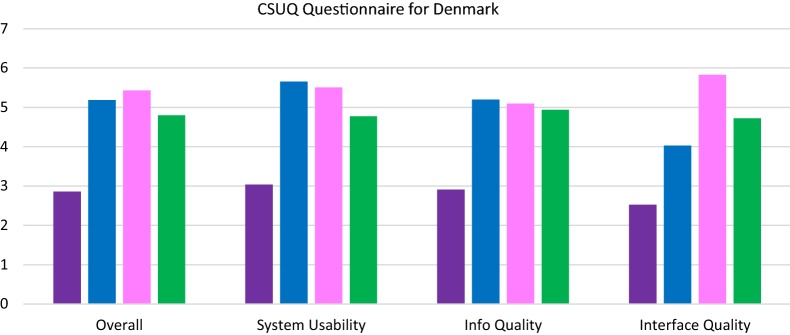
Pulso system and wristband system. Trilogis-Monsenso system and wristband system. Patient-based evaluation of the Trilogis-Monsenso and the PULSO system measured using the CSUQ questionnaire

**Fig. 6:**
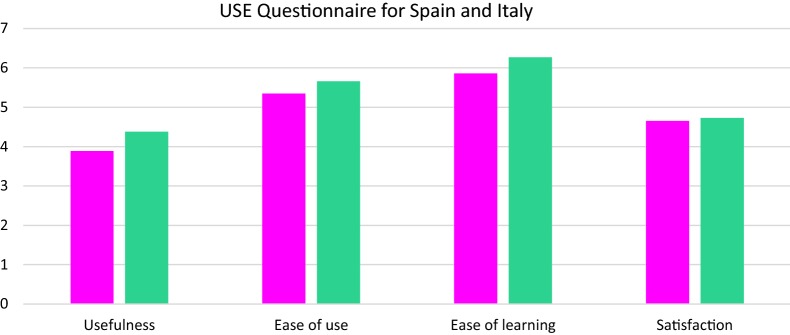
Pulso system and wristband system. Trilogis-Monsenso system and wristband system. Patient-based evaluation of the Monsenso and the MoodRecord system measured using the USE questionnaire

Patient-based evaluations in Italy and Spain: The smartphone-based systems and wristband system were evaluated as a combined system in these two locations. Data from Italy and Spain could not be combined with data from Denmark, since the patients from Italy and Spain did not evaluate the apps and the wristband system separately. In evaluations according to the CSUQ questionnaire the Trilogis-Monsenso system (Trilogis-Monsenso app and wristband system (Nokia Health)) performed better than the Pulso system (Pulso app and wristband system (FitBit)) in system usability and info quality, but not in interface quality (see Figs. 5, 6). In evaluations according to the USE questionnaire, there was no statistically significant difference between the Pulso system and the Trilogis-Monsenso system in any of the categories, except for the usefulness category favoring the Trilogis-Monsenso system (z = 2.68, p < 0.01) (Figs. 5, 6).

### Clinician evaluated disadvantages and possible suggestions for improvements

Clinicians using the two monitoring systems (the Pulso and the Trilogis-Monsenso systems) a part of the NYMPHA-MD studies evaluated the systems after 4 weeks of use. Table 2 present benefits of the two systems from a clinician point of view.

**Table 2:** Clinicians evaluation of benefits of the PULSO systems and the Trilogis-Monsenso systems and wristband systems

Benefits of the Pulso system	Benefits of the Trilogis-Monsenso system
Different psychological tests were installed	Possibility to evaluate sleep
There was the possibility to recording an audio/video file about the day with emotion recognition	Possibility to create and evaluate personalized parameters
	Notification at a self-chosen time to conduct the self-evaluation

Benefits of the Pulso wristband (FitBit)	Benefits of the Trilogis-Monsenso wristband (Nokia Health)
Did not fill up space on the wrist	It did not need to be charged
Good looking design	

The Pulso system: During the monitoring of mood, the patients had to choose between different face icons, such as surprised, sad, happy, neutral, etc. It was possible to choose more than one facial icon to describe the mood for that particular day. One of the clinicians’ concerns were that these icons in fact reflect feelings rather than mood. Furthermore, choosing the face icon that describes the average mood during a day can be quite difficult. For example, many patients reported that they could feel “surprised” in a short period of time, so this face icon would be of limited use to describe the average mood for that particular day. A different mood scale could perhaps improve the monitoring of mood. The evaluation of activities could be done by choosing one activity among five different types of activities. A concern by the clinicians was that most often, all types of activities will be performed during the day, and therefore it may seem of limited use to evaluate in this manner. A different activity scale could perhaps improve the monitoring of overall level of activity for any particular day. According to the clinicians, the evaluation of social media activities seemed difficult as it is difficult to recall how many likes and comments that have been performed during the day on different social media. In addition, it could be quite time-consuming to register this. Perhaps an advantage could be if the system registered these activities automatically. Furthermore, patients complained that they could not go back in time and evaluate in the Pulso app which was also evaluated as a concern by the clinicians. If one could register a single day or two back in time, it could perhaps be an advantage.

The Pulso wristband (FitBit: The battery should be charged every 5–7 days. Longer battery time could be an advantage.

The Trilogis-Monsenso system: During visualization of graphs presenting reported data, many patients sought after the possibility to combine the different graphs. According to the clinicians’ this could provide a good overview of how the different monitoring items might be inter-connected. Some patients could not see the graph of their registration of sleep (technical problem). During graphical visualization, it would be advantageous if it was possible to turn the phone along (and the graph was shown alongside), especially if the patients wanted to see the graph for a period of 3 months without the need to log on to the web-site. Regarding sleep monitoring, the clinicians’ states that the possibility of entering naps during the day and quality of sleep could also be an advantage.

The Trilogis-Monsenso wristband (Nokia Health): The design of the wristband was generally evaluated low from the patients. Some patients only wore it at night because of the design. A more discreet wristband could perhaps be an advantage. The battery did not need to be recharged, which was perceived as an advantage by the patients.

## Discussion

The NYMPHA-MD study was a EU funded PCP project aiming to provide a set of pilot experimentations implementing mobile e-health services for bipolar disorder treatment in a real-world context to be tested in three European countries. The NYMPHA pilot study was able to recruit 60 patients with bipolar disorder with a high participation rate over a short period of time. Overall, the main finding was that both innovative smartphone-based systems developed by two IT companies selected during the PCP project showed adequate usability and feasibility performance.

In Denmark, the Trilogis-Monsenso system was preferred over the PULSO system. However, in Spain and Italy, there were no significant differences in the evaluation of both systems. These results may be determined by different factors including, but not limited to, differences in evaluation methods or the low statistical power due to the small sample size. In Denmark, the smartphone-based system and the wristband were evaluated separately for each company, whereas the smartphone-based system including the wristbands were evaluated as a total system in Italy and Spain. Thus, details regarding differences in evaluations of only the smartphone-based systems or only the wristbands across countries were not available in the present study. The evaluation of the system integrated by the mobile app and the wrist worn band, or conversely, the evaluation of each one of the components separately might modify the subjective perception and bias the analysis of the evaluators in multiple ways. For example, while the independent evaluation of both components can favor a more detailed and specific analysis of each of them, the evaluation of both components as a single system can place less importance to the parts than to the whole, and incorporate different nuances, such as the level of integration, compatibility or complementarity between both. Taken together this aspect and others could generate a significant variation in the final score. Future studies conducting homogenous evaluation methods and including larger samples could investigate this further.

Importantly, in all three study locations both systems (Trilogis-Monsenso system and PULSO system) showed adequate feasibility, usability, usefulness, and technical stability for the start of future clinical trials although there were differences in patient-based evaluations of the two monitoring systems related to the country of the study. The clinicians in Denmark found the Trilogis-Monsenso system more user-friendly and clinically based compared with the PULSO system. The clinicians in Spain and Italy did not showed preferences for one of the two systems except for the usefulness category favoring the Trilogis-Monsenso system (z = 2.68, p < 0.01). Importantly, long-term clinical trials are needed to investigate the effect of these monitoring systems as well as the usability during longer monitoring periods.

Although the findings of patient-based evaluations of feasibility and usability are included in the present study, we did not have access to information on adherence to each app, wear time of wristband etc. This type of information could improve the relevance and impact of the study. Hopefully, the IT companies will publish data on this aspect subsequently.

## Conclusion

Overall, the main finding was that both systems showed adequate usability and feasibility performance. On the other hand, there were differences in patient-based evaluations of the two monitoring systems related to the country of the study and the wristband systems. Future long-term studies could evaluate all functionalities developed by the companies.
